# Relationship between lightning and ice hydrometeors in thunderstorms over northern India from polarimetric weather radar observations

**DOI:** 10.1038/s41598-026-53222-y

**Published:** 2026-05-20

**Authors:** Dharmadas Jash, R. K. Sumesh, E. A. Resmi

**Affiliations:** 1 National Centre for Earth Science Studies (NCESS), Post Box No. 7250, Akkulam, Thiruvananthapuram, Kerala 695011 India; 2 Department of Atmospheric Sciences, Cochin University of Science and Technology (CUSAT), Ernakulam, Kerala 682022 India

**Keywords:** Climate sciences, Natural hazards, Space physics

## Abstract

In this study we have investigated the relationship between lightning and ice-hydrometeors for thunderstorms over northern India. We have implemented a hydrometeor identification (HID) algorithm on a C-band polarimetric Doppler Weather Radar (DWR) data to get information on the ice-hydrometeors and utilized lightning flash data of both cloud-to-ground (CG) and intracloud (IC) type lightnings, from Indian Lightning Location Network (ILLN). Seven prominent thunderstorm events, comprising a total of 385 radar volume scans, have been used in this study. The study reveals a good spatial association between graupel and lightning flashes. Time evolution of ice-hydrometeors and lightning flashes during thunderstorms are in tandem. It is revealed that, decrease in cloud-top brightness temperature (from INSAT-3DR satellite) precedes lightning occurrence by more than an hour. Event-wise correlations between ice hydrometeor related parameters and lightning flash rates show higher values for IC than CG flashes, but less correlations when all the data are pooled across events, signifying higher inter-storm variability in the relationship for IC. Graupel Count (GC), i.e., the number of radar grid points identified as graupel, showed the highest pooled correlation coefficient value of 0.87 for CG lightning. For IC lightning also, GC has the highest correlation coefficient value (0.74). The pooled correlations are higher for CG lightning for all ice-hydrometeor parameters. The findings support the non-inductive collision mechanism of thunderstorm charging. Lag-correlation analysis (up to 60 min of lag) has been performed. Lag correlation coefficient values greater than 0.65 (for CG lightning at 30 min of lag) in case of certain ice-hydrometeor parameters, strongly indicate that they are potential precursors for lightning and could be used for nowcasting of lightning.

## Introduction

Thunderstorms are hazardous mesoscale weather phenomena that occur mainly due to intense convection over the heated surface and are accompanied by heavy rainfall, lightning, and sometimes hail. They have a spatial extent of a few kilometres to few hundred kilometres and a life span of less than an hour to several hours^1–3^. Thunderstorms are common phenomena across the globe, particularly dominant over the tropical belt (due to strong solar heating) as shown by the lightning climatology from the Tropical Rainfall Measuring Mission (TRMM) satellite^4^ data. Globally, about 16 million thunderstorms occur annually, with the Indian subcontinent experiencing some of the highest frequencies due to its favourable thermodynamic and geographic conditions. India’s unique position within the monsoon domain, its extensive land–sea boundaries, complex topography, and seasonal heating contrast make it especially vulnerable to recurrent thunderstorm and lightning activity. In the case of Indian subcontinent, most of the severe thunderstorms occur during the pre-monsoon (March–April–May) season^5^. The topography of the land surface plays a crucial role in modifying atmospheric convective motions, thereby influencing thunderstorm development over a region^6,7^. In mountainous areas, orographic lifting of air parcels acts as a key mechanism that provides the necessary vertical displacement to trigger conditional instability and enhance the convective available potential energy (CAPE) essential for thunderstorm formation^8^. Over India, studies^9–13^ have identified major thunderstorm/lightning hotspot regions, utilizing long period data from IMD stations, TRMM satellite data and ground-based lightning detection network data. Thunderstorms have significant implications for agriculture, aviation, power transmission, human safety, and socio-economic systems. Across India, between 1500 and 2800 deaths occurred annually due to thunderstorms over the period 2001–2017^14^.

Lightning occurrence associated with these thunderstorms, is among the most spectacular and hazardous display of nature. Despite decades of research^15–22^, the microphysical processes that govern its initiation and propagation remain a challenging problem in atmospheric science. It is still not fully understood how such huge amount of electric field is generated within these storms. The most accepted charging mechanism of thunderclouds is the non-inductive charging mechanism, which basically involves charge separation during rebounding collisions between graupel and ice-crystals. Graupels are ice particles with diameter of 2–5 mm, which grow mainly due to riming process i.e., collection of supercooled water droplets onto the surface of ice crystals and subsequent freezing. This charging mechanism does not require any pre-existing electric field. On the other hand, another mechanism viz, Inductive or polarization charging, results from rebounding collisions in the presence of an electric field, which polarizes the particles by forcing ions of opposite sign to accumulate at opposite sides, and charge may be transferred between particles when they collide and separate^22,23^. The non-inductive charging theory came forward because of several pioneering laboratory studies^24–27^, which have shown that such collisions can in fact generate significant amount of charge separation capable of producing strong electric field leading to lightning discharge. After collision, the graupel usually gain negative(−Ve) charge and ice-crystals gain positive (+ Ve) charge. But the sign of the charges on these particles depends on several factors such as—air temperature, cloud water content (CWC), rime accretion rate (RAR) of the graupel^18,24,26,27^. As per the laboratory study by Takahashi^27^, the graupel gain positive charge at temperatures higher than − 10°C regardless of the CWC. On the other hand, laboratory study by Jayaratne et al.^24^ showed that graupel gain negative charge even at temperatures higher than − 10°C at sufficiently less CWC. This temperature, at which the charge on graupel changes sign is known as the reversal temperature. The sign of this transferred charge depends also on the cloud droplet spectra^28^. Since, aerosols play direct role in the cloud droplet spectra, they influence the sign of this transferred charge. Aerosols can indirectly modify RAR, which has direct link to the sign of transferred charge^18^. Several studies have demonstrated the role of aerosols in thunderstorm charge structures, both from observations^29–33^, as well as numerical simulations^34–37^. Inverted polarity charge structure of thunderstorms has been linked to the aerosols in the atmosphere^30–33,38^. As a result of this charge separation, a dipolar charge structure is generated with negative charges centred around − 10°C level and the positive charges centred around − 40°C level^23^. Occasionally another secondary positive charge centre is seen at lower levels near 0°C level forming a tripolar charge structure^39^. Stolzenburg et. al.^40^ obtained detailed charge structures within thunderstorms from data obtained via 50 balloon soundings through such storms. Such charge structure leads to positive electric field (i.e. in upward directed).

It is imperative to validate these charging theories that have been put forward from laboratory studies, in the real atmosphere. Several studies have shown relationships between storm parameters and lightning flash rate, e.g., Price and Rind^41^ have linked lightning flash rate with the cloud top height and to the thunderstorm updraft speed. Deierling and Petersen^42^ have shown a good correlation between total lightning flash rate and the storm volume having updraft greater than 5 m s^−1^ within the charging zone (i.e. at temperature < − 5°C). Numerous studies have attempted to address the relationship between ice hydrometeors (graupel and ice crystals) and lightning as suggested by the non-inductive charging mechanism. A simple calculation by Blyth et al.^43^ has shown that lightning frequency (f) is proportional to the product of the precipitation rate (p) of graupel and the upward mass flux of ice crystals (F_i_), the values of these quantities being taken at the top of the charging zone. Later, Latham et al.^44^ have shown that this relationship depends on the glaciation mechanism (primary nucleation or Hallett-Mossop multiplication) involved. Deierling et al.^45^ have shown that time series of calculated hydrometeors i.e., precipitable and non-precipitable ice follows the overall pattern of total lightning and for most times show a strong correlation. Using polarimetric DWR data Deierling et al.^46^ has shown there exist strong relationships between mass fluxes of precipitating and non-precipitating ice hydrometeors and lightning flash rates. A study over Japan by Hayashi et al.^47^ has shown that there are very good correlations between different parameters related to ice-hydrometeors and lightning flash rate. But, when it comes to the Indian region, such studies are rare. Utsav et al.^48^ has studied the relationship between storm properties and lightning utilizing X-band radar and lightning location network observations.

Even though the broad aim of these earlier studies (e.g.,^46–49^) are similar to our study, which is understanding the relationship between lightning and ice hydrometeors, but they differ significantly in terms of methodologies used as well as regional differences. Carey and Rutledge^49^, Deierling et al.^46^ have studied the relationship between lightning and ice hydrometeors in terms of mass fluxes of graupel and ice-crystal, whereas our study involves several parameters related to different ice-hydrometeors (viz., graupel, ice crystal, vertically oriented ice, aggregates). Carey and Rutledge^49^ have conducted their study with thunderstorm observations over Tiwi Islands, situated in northern Australia and Deierling et al.^46^ have performed their study with thunderstorm observations over Northern Alabama/Colorado, US, which are having completely different latitude/longitude and the thunderstorm dynamics may be different from that over the Indian region. Over the Indian region, Utsav et al.^48^ have studied the relationship between lightning and storm intensity parameters (such as 35 dBZ Echo Top Height crossing 9 km), but their study did not incorporate any ice-hydrometeors, which have direct link to lightning occurrence. Hence, it is worthwhile to study the relationship between ice-hydrometeors and lightning occurrence over the Indian region, given the significant impact of lightning on the society. Also, none of these studies have quantified the spatial relationships between the lightning flash and ice-hydrometeors.

In this study we have investigated the relationship between lightning occurrence and ice-hydrometeors in the thunderclouds. Several ice-hydrometeor related parameters have been calculated and their correlation with lightning have been explored. Possibilities of lightning nowcasting using these parameters as precursors, have been discussed. The paper is organized as follows—apart from the introduction (Section “Introduction”), the data used in the study and methodologies applied are described in Section “Data and methodology”; results and discussions are presented in Section “Results and discussions”; and Section “Summary and conclusions” summarises the major findings of the study.

## Data and methodology

For this study we have used data from (i) Indian lightning location network (ILLN) for lightning flash, (ii) C-band dual polarimetric Doppler weather radar (DWR) for different polarimetric variables, and (iii) INSAT-3DR satellite for cloud-top infrared brightness temperature. A brief over of these data and instruments are given below.

### Dual-polarization Doppler weather radar data

**Fig. 1:**
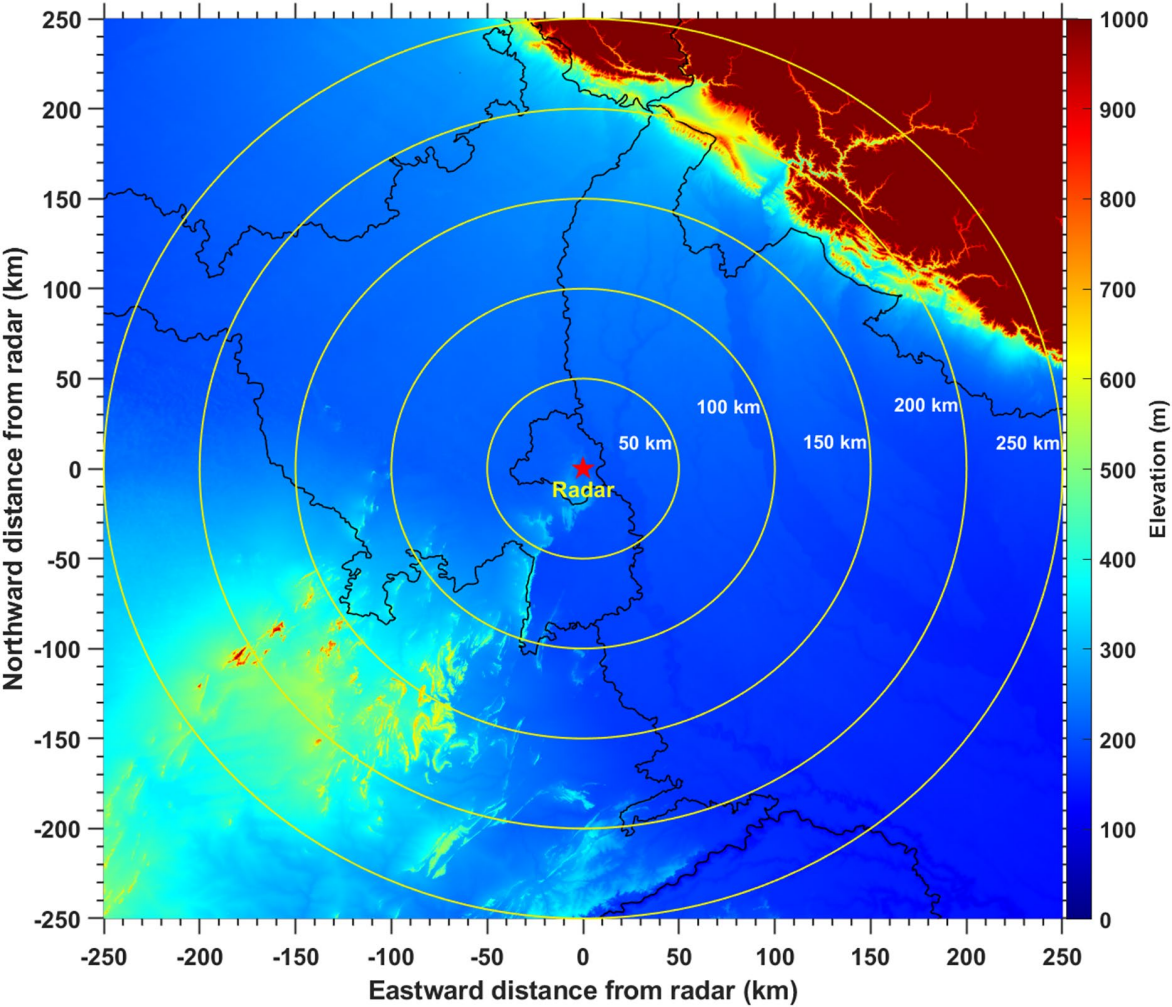
Location of the Doppler weather radar at Delhi and terrain heights of the surrounding region are shown. Red star at the centre denotes the DWR location and circles represent the radial distances from the DWR.

In this study, we have utilized data from C-band Doppler weather radar (DWR) installed at Delhi (28.5898N, 77.2219E, 253 m above mean sea level) operated by India Meteorological Department (IMD). It is an active remote sensing instrument which sends electromagnetic pulses at a frequency of 5.625 GHz. The radar scans the atmosphere at 10 elevation angles (0.5°, 1°, 2°, 3°, 4.5°, 6°, 9°, 12°, 16° and 21°) with a radial resolution of 300 m and azimuthal resolution of 1°. It has a pencil beam of 1° beam width. The radar scans the surrounding atmosphere within a radius of about 250 km. The DWR takes around 10 min to generate one full volume scan and stores data of each sweep (i.e., scan at one fixed elevation) in separate files. The radar provides base products such as reflectivity at horizontal polarization (Z_h_), differential reflectivity (Z_dr_), differential propagation phase (Φ_dp_), cross-correlation (ρ_hv_), radial velocity (V_r_) and Spectral width(σ). Z_dr_ is the difference between reflectivities (in decibel) at horizontal and vertical polarization. Φ_dp_ is the phase difference between the horizontally and vertically polarized pulses. Certain quality-control measures have been applied to the radar dataset and can be found in Jash et al.^50^. Specific differential phase (K_dp_) estimation is also explained in this paper. This DWR data is available from IMD website https://radarapi.imd.gov.in/ on request basis. The available data of all polarimetric variables from the DWR observations for seven thunderstorm events during pre-monsoon (Mar–May) season of 2021 have been used in this study. The radar site and its surrounding terrain are shown in Fig. 1.

### Lightning location network data

Indian Lightning Location Network (ILLN) is a system established by the Indian Institute of Tropical Meteorology (IITM), Pune under the Ministry of Earth Sciences (MoES), to detect location and time lightning occurrences across India. The network consists of several sensors strategically installed across different parts of India. The details of the network and validation of its data can be found in Biswasharma et al.^51^ and Srivastava et al.^52^. These sensors are manufactured by Earth Networks (https://www.earthnetworks.com). When there is a lightning strike, it emits electromagnetic radiation across a wide range of spectrums. The sensors receive this radiation in very low frequency (VLF), low frequency (LF), and high frequency (HF) radio bands to detect lightning. The network can differentiate between IC and CG lightning. If lightning occurs, multiple sensors capture the waveforms and transmit them to the central server. The server then applies the time-of-arrival algorithm (TOA;^53^) to precisely obtain location (latitude, longitude, altitude) of the IC/CG lightning. The network has a lightning detection accuracy of ~ 100 m. This data has been used in several studies^32,48,54–56^ proving its usefulness and reliability. Lightning strike data for both CG and IC types are considered for this study. One should keep in mind that the detection efficiency of IC lightning is usually smaller than that of CG lightning, mainly due to less intense waveform coming out of the former. Even though there is no systematic study over the Indian region to quantify this detection efficiency for IC vs CG lightning, some studies have highlighted that this network has detection efficiency of 50% and 90% for IC and CG lightning respectively^30,54,57^.

Identification of the thunderstorm events have been done using the lightning flash data from ILLN. A region of 200 × 200 km has been considered as the study region centred at the radar location. Time series of lightning flash data over this region were considered to identify the prominent thunderstorms events. This way we have identified seven prominent thunderstorm events as shown in Table 1. The analysis in this study is done for the data during these events. Out of these seven events, the events on 06/05/2021 and 09/05/2021 are having maximum number of CG flash and also the event on 09/05/2021 displays multiple peaks in lightning activity. Hence these two events are considered for the time series analysis.

**Table 1:** Details of the thunderstorm events considered in this study.

Sl. No	Date	Start time (UTC)	End time (UTC)	Duration (HH:MM)	CG flash count	IC flash count
1	09/03/2021	10:00	18:45	08:45	501	2211
2	23/04/2021	00:00	08:00	08:00	797	3460
3	06/05/2021	06:00	15:00	09:00	1728	3718
4	09/05/2021	07:00	20:00	13:00	1157	1790
5	11/05/2021	10:00	19:00	09:00	405	1326
6	13/05/2021	00:00	06:00	06:00	127	725
7	31/05/2021	16:00	23:55	07:55	945	5448

### INSAT-3DR satellite data

Cloud-top Infrared brightness temperature data has been used as a proxy for the cloud top height. This data was obtained by INSAT-3DR satellite^58^, which is a multi-purpose geosynchronous spacecraft and provides data with spatial resolution of 4 × 4 km and temporal resolution of 30 min of mesoscale phenomena in the visible and infrared (IR) spectral bands (0.55–12.5 μm) over the Indian region. The data is freely available through the https://www.mosdac.gov.in/ server. This data has been used to see if changes in cloud top temperature could be used as a precursor for lightning activity.

### Hydrometeor identification and related parameters

A hydrometeor identification algorithm by Dolan et al.^59^ is used to identify types of hydrometeors present at different height levels within a convective system. This is a fuzzy logic-based algorithm in which fuzzification of the inputs is done by calculating values of the membership functions corresponding to each fuzzy set (here, different hydrometeor types). Then fuzzy score for each fuzzy set is calculated using Equation-1. This step is called aggregation. Then defuzzification is done by choosing the fuzzy set corresponding to the maximum fuzzy score i.e., hydrometeor with the highest fuzzy logic score is the most probable hydrometeor type at that grid point within the radar scan volume.

1 $$\mu_{i} = \left[ {\frac{{W_{{Z_{dr} }} \beta_{{Z_{dr} ,i}} + W_{{K_{dp} }} \beta_{{K_{dp} ,i}} + W_{{\rho_{hv} }} \beta_{{\rho_{hv} ,i}} }}{{W_{{Z_{dr} }} + W_{{K_{dp} }} + W_{{\rho_{hv} }} }}} \right]\beta_{T,i} \beta_{{Z_{h} ,i}}$$

2 $$\beta = \frac{1}{{1 + \left[ {\left( {\frac{x - m}{a}} \right)^{2} } \right]^{b} }}$$

Here, μ_i_ is the fuzzy logic score for the i^th^ hydrometeor type. β_j,i_ is the membership function for i^th^ hydrometeor types and j^th^ variable (Equation-2). W_j_ is the weight factor for the j^th^ variable. The values of these membership function parameters and the weights are taken from Dolan et al.^59^, which they obtained for simulations at C-band. Five variables viz. Z_h_, Z_dr_, K_dp_, ρ_hv_ and temperature (T) are used to calculate the fuzzy score. Here, K_dp_ is the slope of range profile of Φ_dp_. It is an important parameter for meteorological applications as it is closely related to rain intensity. Though the estimation of K_dp_ seems quite simple, it requires pre-processing of Φ_dp_ range profiles before calculating the slope, as Φ_dp_ is known to be a very noisy parameter particularly in regions with low rain rates and the process of differentiation increases this noise even further. To tackle this, we have applied a low-pass butterworth filter of order 10 with a cut-off scale of 2 km to reduce the statistical fluctuation but keeping the overall features intact. The use of such filters can be seen in earlier studies^60,61^. Seven types of hydrometeors have been considered here viz. drizzle (DZ), rain (RN), ice crystals (CR), aggregates (AG), low-density graupel (LDG), high-density graupel (HDG), and vertically oriented ice (VI). Temperature for the HID scheme has been obtained from ECMWF Reanalysis v5 (ERA5) data, which is widely used in weather and climate studies^62,63^. This data is available at the website https://cds.climate.copernicus.eu/datasets/reanalysis-era5-pressure-levels.

We have followed methodologies similar to that of Hayashi et al.^47^. Several ice-hydrometeor related parameters have been estimated and their relation to the lightning occurrence has been analyzed in this study. But our methodology differs from them in certain aspects, such as, we have incorporated vertically oriented ice, which is directly influenced by the presence of strong electric fields in thunderstorms. Also, we have considered ice crystal in the charging zone (CRC_CZ) and storm intensity parameter (ETH9C). These parameters are as follows.

- GC—Graupel Count, i.e., the number of radar grid points identified as graupel.
- CRC—ice crystal count, i.e., the number of radar grid points identified as ice crystal.
- CRC_CZ—Ice-crystal count relevant to the charging zone (i.e., above − 25°C level).
- VIC—Vertical ice count, i.e., the number of radar grid points identified as vertically oriented ice.
- ALICC—All ice count, i.e., the number of radar grid points identified as any type of ice hydrometeor.
- C35IC—number of radar grid points identified as any type of ice hydrometeor and having reflectivity (Z) greater than 35 dBZ.
- C40IC—number of radar grid points identified as any type of ice hydrometeor and having reflectivity (Z) greater than 40 dBZ.

Besides these ice-hydrometeor-related parameters, we have estimated a storm-intensity-related parameter, viz. ETH9C, which is defined as the count of the locations over which the 35 dBZ echo top height (ETH) is greater than 9 km. As shown by Utsav et al.^48^, 35 dBZ ETH crossing 9 km height correlates well with lightning occurrence. Hence, we have incorporated this ‘ETH9C’ parameter to assess whether the ice-related parameters have better correlations with lightning as compared to the storm-intensity-related parameter.

All these counts have been calculated for the 200 × 200 km region, considered for the study as mentioned earlier.

These specific parameters are estimated keeping in mind that as per the non-inductive charging mechanism the presence of graupels and ice crystals are the most important hydrometeors. Presence of vertically oriented ice (VI) signifies the presence of strong electric fields which align them vertically, hence they are also important for studying the lightning occurrence.

## Results and discussions

Thunderstorm initiation requires a favorable combination of three primary ingredients: moisture, instability, and a lifting mechanism^64^. While atmospheric moisture and instability provide the necessary energy through convective available potential energy (CAPE), it is the lifting mechanism that triggers the release of this energy by overcoming the convective inhibition (CIN) energy at the lower level and lifting the air parcels beyond the level of free convection (LFC). Among the various lifting mechanisms, low-level wind convergence is widely recognized as a fundamental driver of thunderstorm formation^65–67^.

**Fig. 2:**
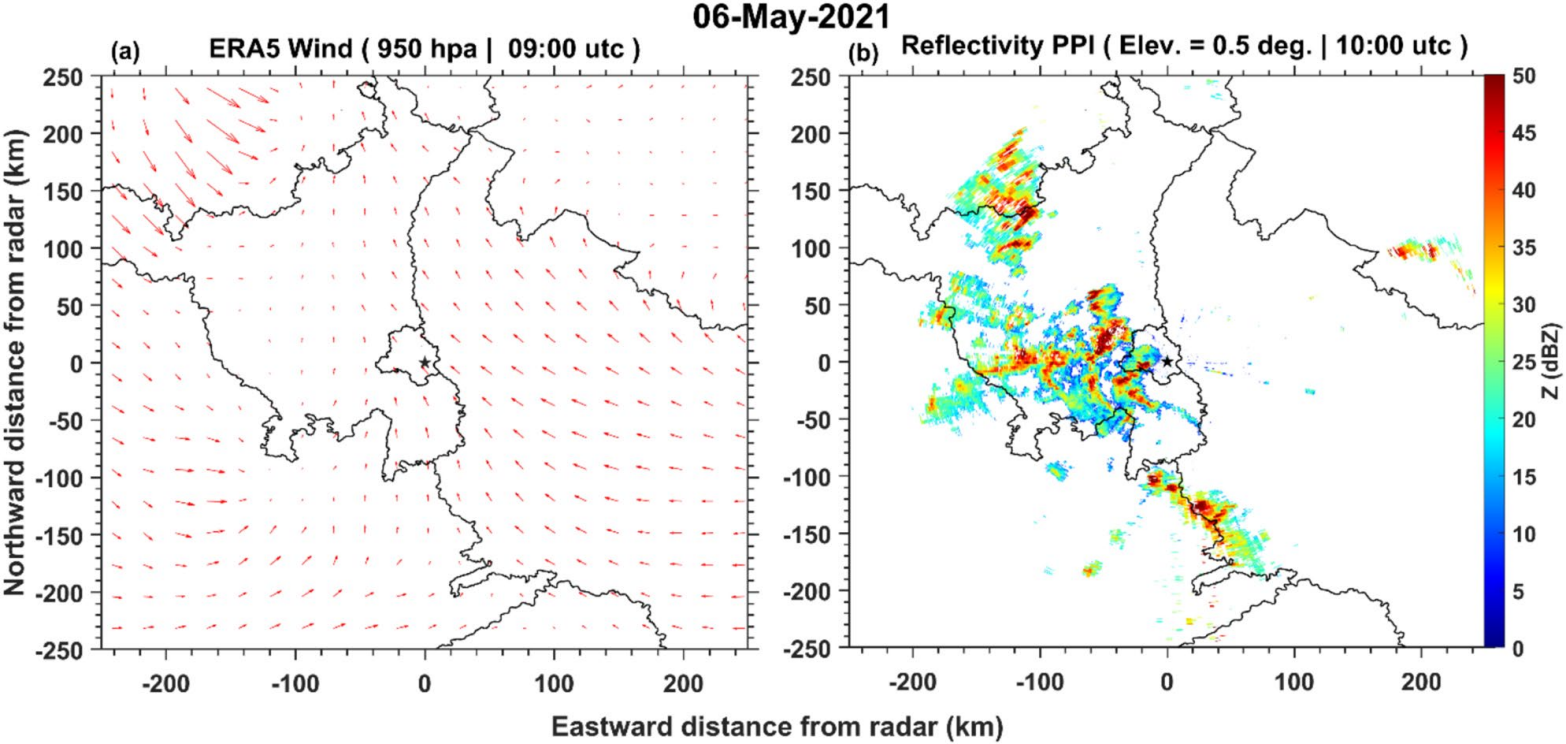
Thunderstorm event on 06-May-2021 over Delhi. (**a**) vector plot of horizontal wind from ERA5 data. (**b**) Radar reflectivity from C-band DWR. Black star at center represents the radar location.

Figure 2 shows the low-level wind conditions prevailed during the initiation phase of the thunderstorm on 06-May-2021. Figure 2a shows the vector plot of the horizontal wind at 09:00 UTC at 950 hpa level i.e., at lower level of the atmosphere. This plot shows a clear mesoscale convergence of horizontal wind at near surface levels. This low-level convergence of wind has created a forced upward motion due to mass continuity and provided the required trigger mechanism to overcome the CIN. In Fig. 2b, we see the development of the subsequent thunderstorm as depicted in plan position indicator (PPI) diagram of the radar reflectivity at 0.5° elevation angle at 10:00 UTC (i.e., 1 h later than the wind observation). Strong reflectivity values (dark red patches) represent presence of bigger raindrops or ice particles due to the presence of strong updraft, which can sustain these particles in air for sufficiently longer times to grow to bigger size via collision-coalescence (for rain drops) or riming (for ice particles) processes.

### Spatial pattern—graupel vs lightning

**Fig. 3:**
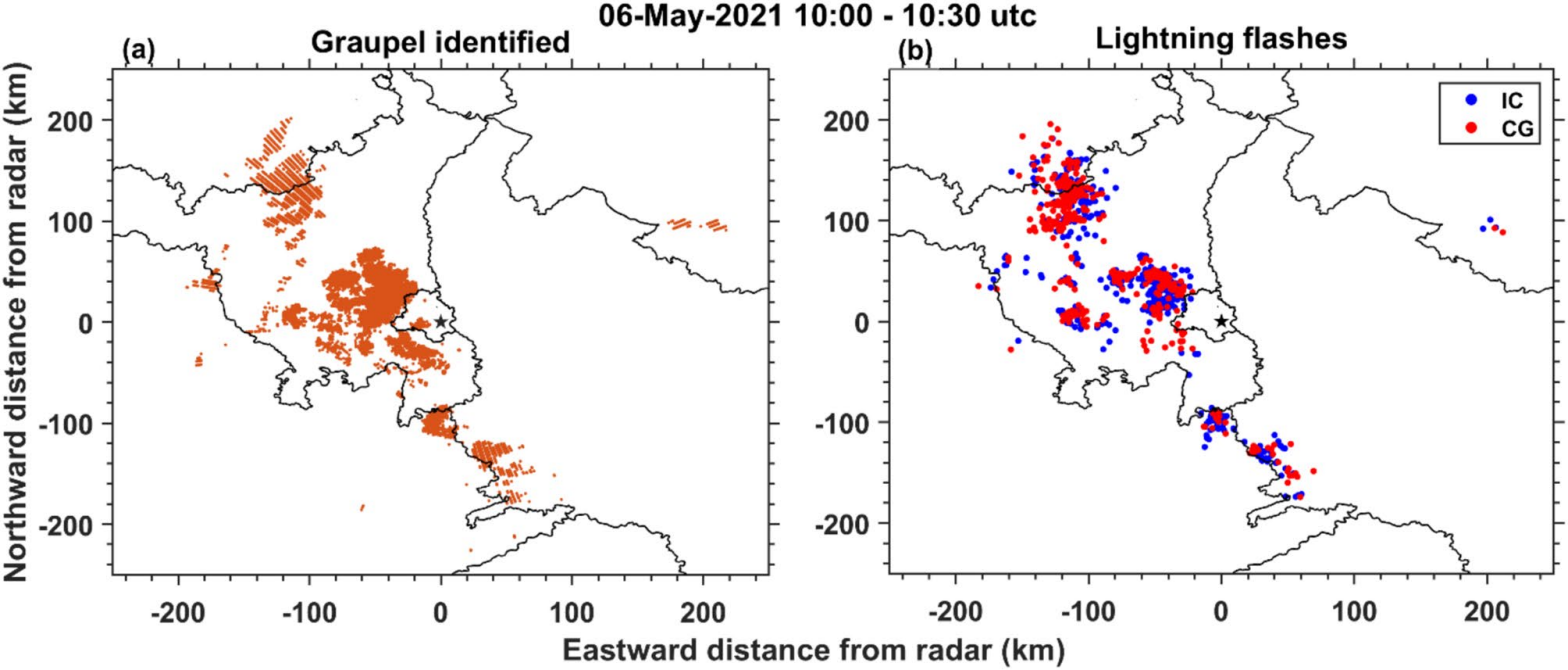
(**a**) Graupel identified over the thunderstorm region and (**b**) the observed CG (red dots) and IC (blue dots) types of lightning by the ILLN during 10:00–10:30 UTC for the thunderstorm event on 06-May-2021.

To understand the link between ice hydrometeors and lightning, we have attempted to identify their relationship in terms of both their spatial patterns and temporal evolution. Similarity between the spatial patterns of graupel and lightning occurrence during the mature phase of the storm between 10:00 and 10:30 UTC on 06-May-2021 has been depicted in Fig. 3. Over a particular location (i.e., a given lat-lon point), if graupel (either HDG or LDG) is identified at any grid point along the vertical, then that location has been marked on the map. Thus, the locations where graupel has been identified are shown in Fig. 3a. On the other hand, we have shown lightning occurrence during this period in Fig. 3b. Both cloud-to-ground (CG) and cloud-to-cloud (CC) type lightnings have been shown here. Remarkable similarities between the patterns of graupel and both CC and CG type of lightning are observed.

**Fig. 4:**
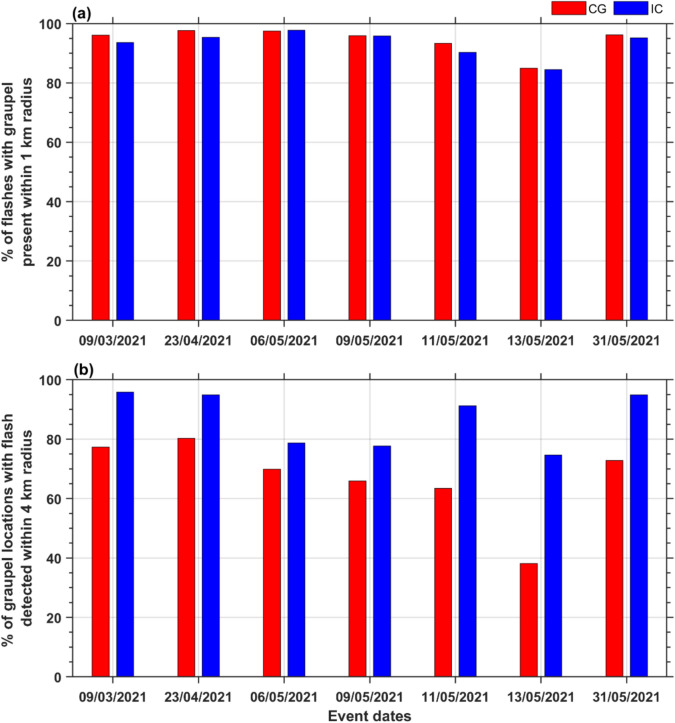
(**a**) Percentage of flashes with graupel identified within 1 km radius and (**b**) percentage of graupel locations that have associated flash detected within 4 km radius for all the seven thunderstorms. Red and blue bars correspond to CG and IC lightnings respectively.

To quantify this similarity in spatial patterns of graupel and lightning, we have done further analysis. To understand how closely they occur, we have calculated two estimates—(i) percentage of flashes with graupel identified within 1 km radius and (ii) percentage of graupel locations that have associated flash detected within 4 km radius, for all the seven thunderstorms. The results are shown in Fig. 4. As seen in Fig. 4a, for a very high proportion of lightning flashes, graupel has been identified within 1 km radius of the flash location, for all seven events. Both for CG and IC flashes, these values are greater than 95% (except for 13/05/2021). On the other hand, as seen in Fig. 4b, the percentage of graupel identified locations, which have associated flash detected within 4 km radius, shows greater variability across events. For CG flashes, the percentage ranges from approximately 38% to 80%, whereas for IC flashes it varies from about 74% to 96%. IC flashes consistently exhibit a higher spatial association with graupel than CG flashes across all events. The choice of 4 km radius here is ad hoc, but it is justified based on the fact that lightning strike can occur more than 10 km away from the main convective center^68,69^. These findings highlight an important asymmetry in the spatial relationship. While most lightning flashes occur near graupel, only a subset of graupel regions produce lightning flashes within the chosen spatial threshold (i.e., 4 km). This suggests that although presence of graupel is a necessary condition for electrification, it is not sufficient for lightning production everywhere within the storm. Additionally, the consistently higher IC association compared to CG flashes indicates that graupel regions are more closely related to in-cloud electrical activity. Overall, the results demonstrate a robust spatial linkage between graupel and lightning activity across all the events, supporting the fact that graupel regions play a critical role in thunderstorm electrification.

### Temporal evolution—hydrometeor parameters vs lightning

Time evolution of different hydrometeor related parameters, which were explained in previous section, have been compared with the time evolution of the lightning flash rates for both CG and IC types of lightning to assess the relationship between them. Lightning flash rate has been calculated per 10 min, because the DWR completes a full volume scan every 10 min and saves the data. Only those flashes that occurred within the study region (i.e., 200 × 200 km box centred at the DWR) have been counted. Here, we have shown the results for two thunderstorm events.

**Fig. 5:**
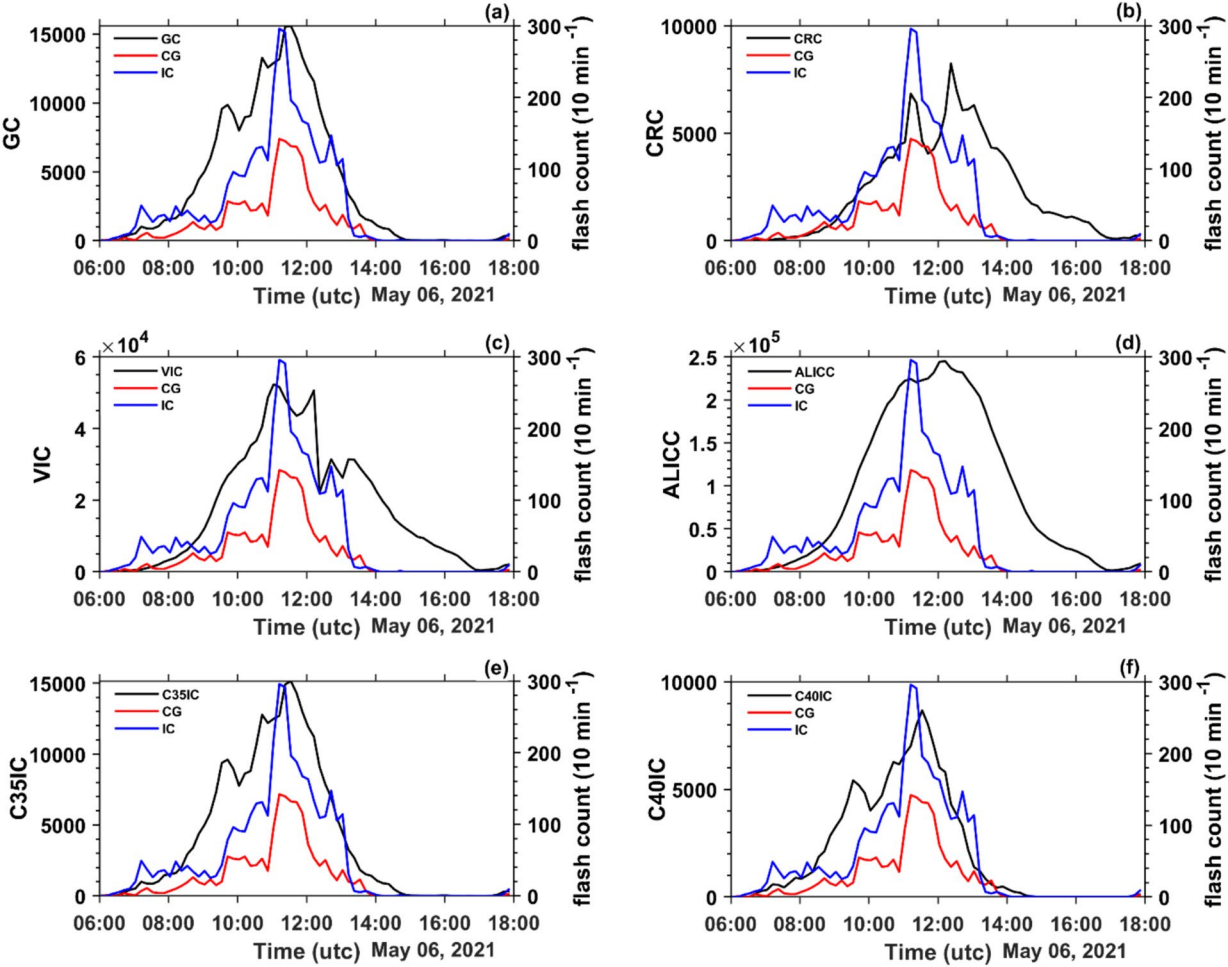
Time series of lightning flash rates for CG (red curves) and IC (blue curves) lightning and different hydrometeor parameters (black curves) during the thunderstorm on 06-May-2021. In each panel left y-axis shows the hydrometeor parameter values, and right y-axis represents the lightning flash rates for both CG and IC types. (**a**) GC and flash rate, (**b**) CRC and flash rate, (**c**) VIC and flash rate, (**d**) ALICC and flash rate, (**e**) C35IC and flash rate, and (**f**) C40IC and flash rate.

In Fig. 5, these times series have been compared for the thunderstorm event on 06-May-2021. Time evolution of lightning flash rates and all the hydrometeor parameters are in tandem with each other. As per non-inductive charge separation, one would expect the peak in lightning to occur at a later time than that of the hydrometeor parameters and the peak-to-peak correspondence in this study could be attributed to the use of domain averaged data for the time series plot. Even though, the peaks occur at similar times, as we will see in the subsequent analysis that there exists good lag correlation between them.

We have already seen the association between graupel and lightning in terms of similarities in the spatial patterns of graupel and lightning occurrence. Here in Fig. 5a, we see that the time evolution of GC (graupel count) and lightning flash rates are almost identical with their peaks at the same time. In Fig. 5b, we see that the peak in the CRC (ice crystal count) occurs at a later time than that for the flash rates, even though there is a secondary maxima that coincides with that of the flash rates. In Fig. 5c, though the peak of the VIC (vertical ice count) occurs at the same time as that for the flash rates, but subsequently the VIC decreases gradually on contrary to the flash rates which decreases sharply. This may be due to the fact that the electric field values required for the vertical alignment of these ice crystals is less than the field values required for the lightning initiation. Figure 5d shows that the peak in the ALICC (all ice count) occurs at a later time than that for the flash rates. Also, the ALICC values remain high even though lightning flash rates becomes zero. This suggests that all the ice hydrometeors do not take part actively in the charge separation mechanism. Figure 5e–f show that the time evolution of C35IC and C40IC are very much similar to that of the GC (Fig. 5a). This is due to the fact that higher reflectivity values (i.e., greater than 30 or 40 dBZ) at higher levels are due to the bigger ice hydrometeors such as graupel. Our results are in line with the findings of Deierling et al.^45^ in which the authors have shown that time evolution of precipitable ice fraction and lightning flash rate are in tandem during a multicell thunderstorm.

**Fig. 6:**
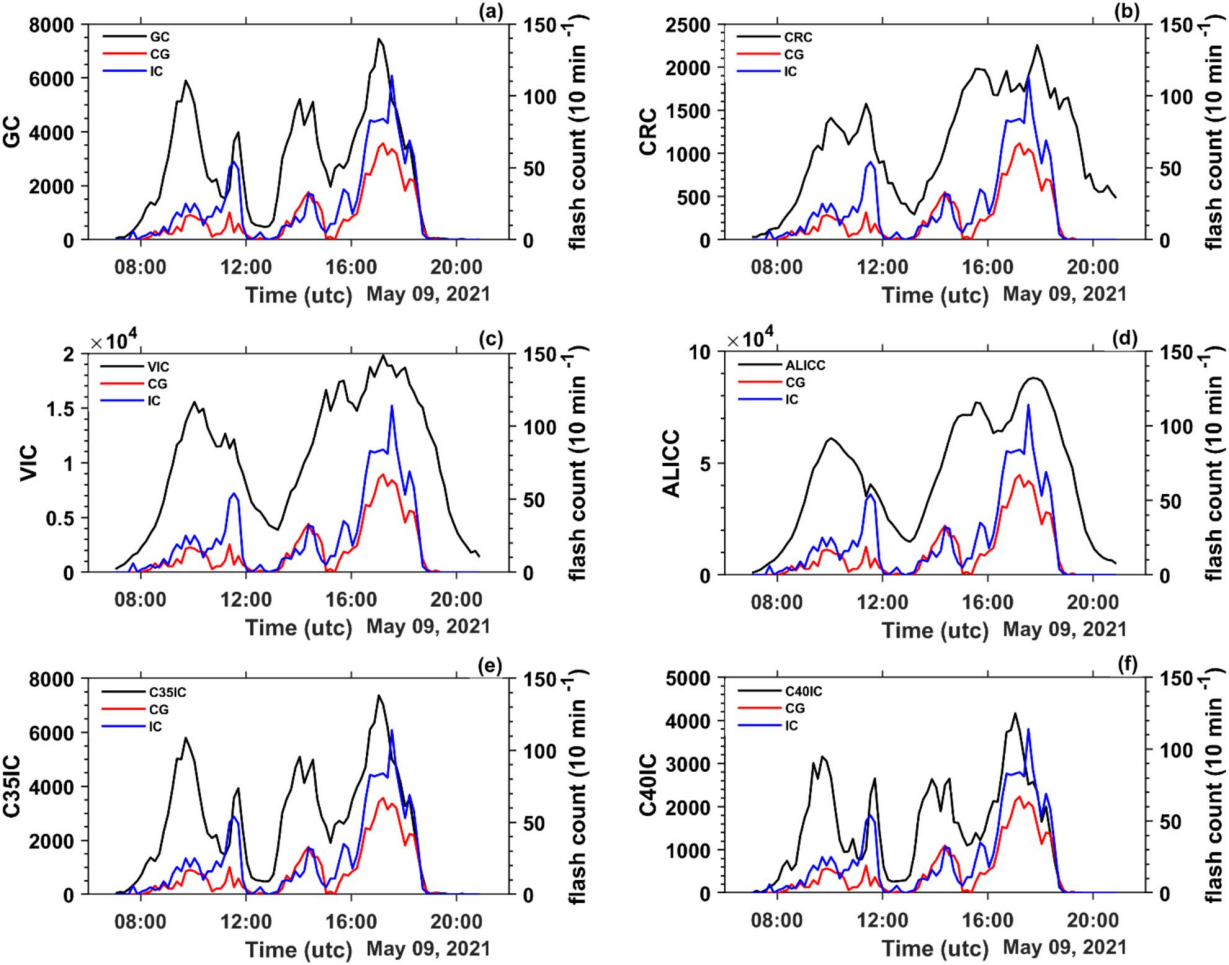
Same as in Fig. 5 but for the thunderstorm event on 09-May-2021.

Similar comparison of time evolutions of hydrometeor parameters and flash rates for another thunderstorm event on 09-May-2021, have been shown in Fig. 6. In this case, we see multiple peaks in the lightning flash rates during the course of the event and the multiple peaks in hydrometeor parameters are also observed. Particularly, time evolution of GC, VIC, C35IC and C40IC follow very closely to that of the lightning flash rates. The highest peak in lightning activity corresponds to the highest peak in the hydrometeor parameters (in all panels of Fig. 6). The initial peak in the hydrometeor parameters (particularly GC) is not fully understood and could be due to build up of initial charges in the clouds until first breakdown. Using a C-band dual polarimetric radar, Carey and Rutledge^49^ have studied a tropical convective complex system to explore the relation between mixed phase ice mass flux and lightning activity. Like our second case (09-May-2021), this storm also showed multiple peaks in CG flash rate. They have also found a similar temporal variation in graupel mass and CG flash rate.

### Correlation between hydrometeor parameters and lightning

**Fig. 7:**
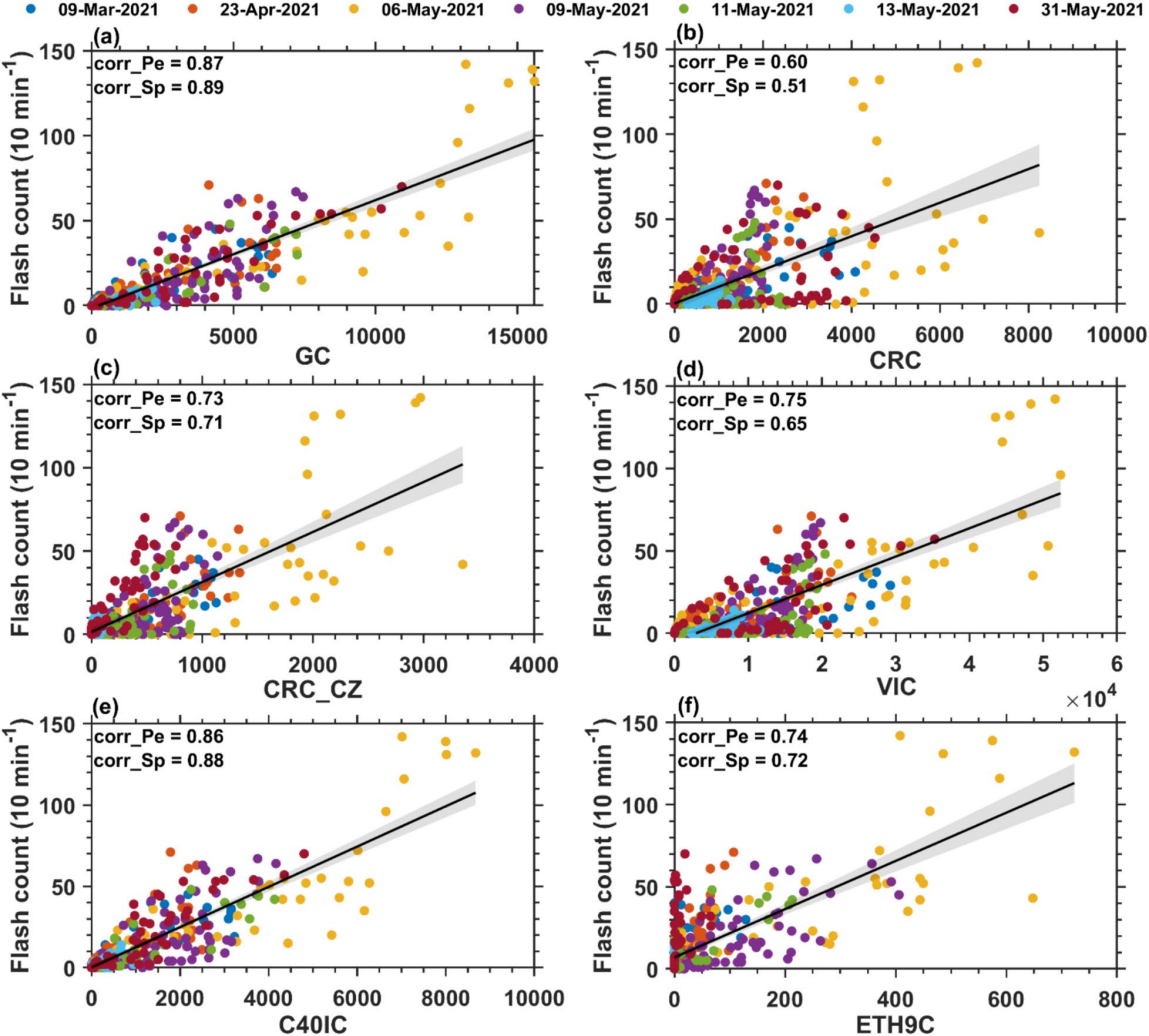
Scatter plot between flash rate of CG type lightning and hydrometeor parameters—(**a**) GC, (**b**) CRC, (**c**) CRC_CZ, (**d**) VIC, (**e**) C40IC, and (**f**) ETH9C. Data taken during all the seven events. Black line represents the linear regression fit between the x-axis and y-axis data. Shaded region is the 99% confidence interval. Correlation values between flash rate and hydrometeor parameters are shown in each panel.

**Fig. 8:**
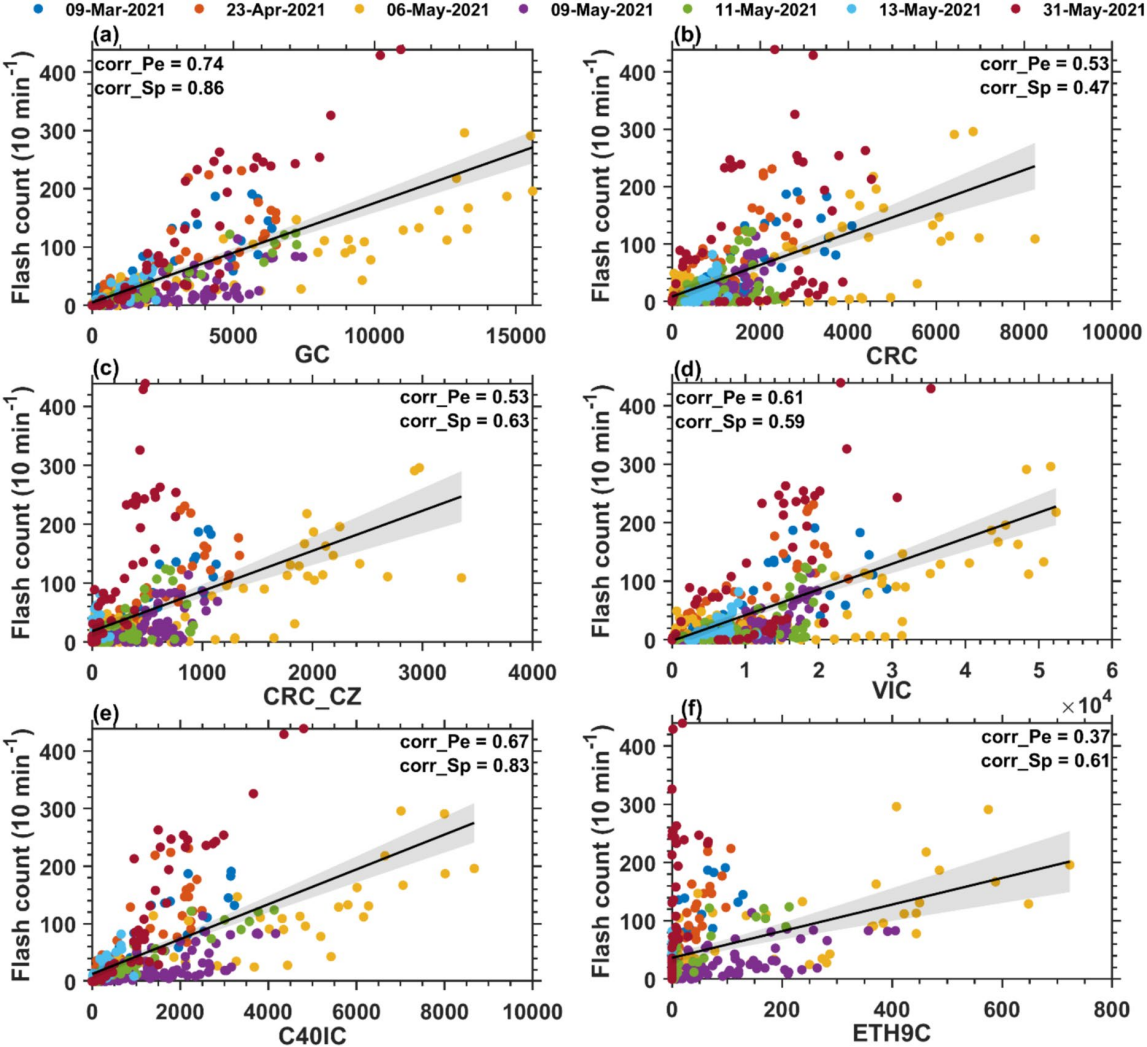
Same as Fig. 7 but flash rates for IC lightning.

Earlier analysis showed that there exists good relationship between ice hydrometeors and lightning occurrence. To quantify this further we have calculated the Pearson and Spearman correlations. Pearson correlation quantifies the linear relationship between two variables and Spearman correlation measures the non-linear but monotonic relationship. the scatter plots between hydrometeor parameters and flash rates of CG and IC lightning have been shown in Figs. 7 and 8 respectively. Each data point in these plots represents data during a single radar volume scan, i.e., hydrometeor parameters have been calculated for each volume scan and, the lightning flash counts have been calculated during the same period (10 min). We have total 385 volume scans form the seven thunderstorm events considered in this study, i.e., there are total 385 data points in each scatter plot. Correlation values between flash rates and hydrometeor parameters have been shown in corresponding scatter diagrams. Linear regression fit (black line) along with 99% confidence interval (shaded region) have been shown in each panel. For both CG and IC lightning, we can see the less scatter in the data points for GC, C40IC and VIC compared to the other parameters indicating a stronger relationship. When we make a comparison between the CG and IC types, for a given parameter (e.g., GC), the scatter is more in case of IC compared to CG lightning. Though the overall scatter in the data is more.

in case of IC, but event wise scatter in the data is less, e.g., the data for the event 06/05/2021 (yellow circles) and 31/05/2021 (red circles) are well correlated for the individual cases, but the slope of their relationships are very different and will lead to poor overall correlation. To assess this clearly an analysis on event wise correlations has also been done here.

**Fig. 9:**
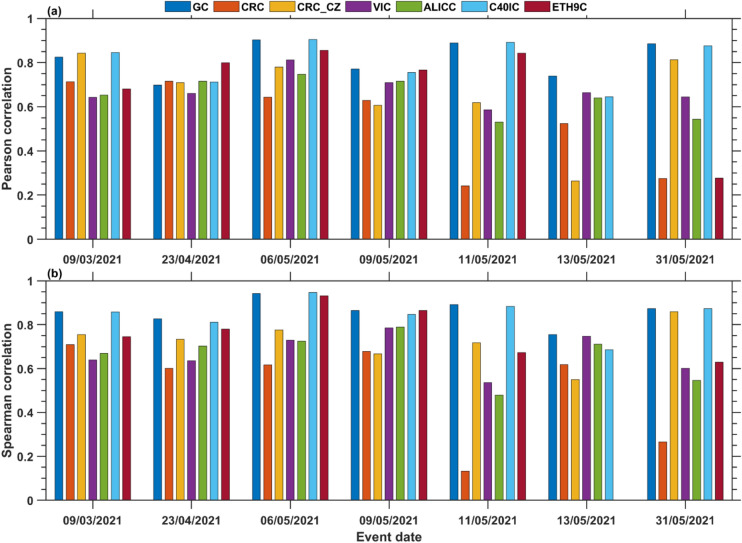
Event-wise correlation values between ice-hydrometeor parameters and lightning CG flash rate. Different coloured bras represent different parameters. (**a**) Pearson correlation and (**b**) Spearman correlation.

Event wise correlations between hydrometeor parameters and lightning flash rate for the seven events have been presented in Fig. 9 for CG lightning. GC consistently exhibits high correlation (~ 0.7–0.9) with CG flash rate across events which indicates that presence of graupel is strongly associated with CG lightning production. For CRC, correlation is generally moderate (~ 0.5–0.7) for most of the cases and suggests that small ice crystals alone are not the dominant driver of CG flashes. For CRC_CZ, correlation improves compared to the total CRC and the variability in the correlation across events is much less. This indicates that location of crystals within the mixed-phase charging region is more important than their total abundance. VIC shows correlation values of ~ 0.6–0.8 consistently. ALICC has moderate correlation values of ~ 0.5–0.7. C40IC shows correlation values similar to that of GC. For ETH9C, correlation values in the range ~ 0.3–0.85 are seen. In most cases (except 23/04/2021) the correlations are less than that of the GC. Also note that the bar corresponding to ETH9C is missing on 13/05/2021 as there was no location over which the 35 dBZ ETH crossed 9 km for this event, even though lightning activity was observed. These results clearly demonstrate that the relationships between microphysical proxies such as GC (graupel count) and lightning activity are stronger compared to that between storm intensity proxies such as ETH9C and lightning.

**Fig. 10:**
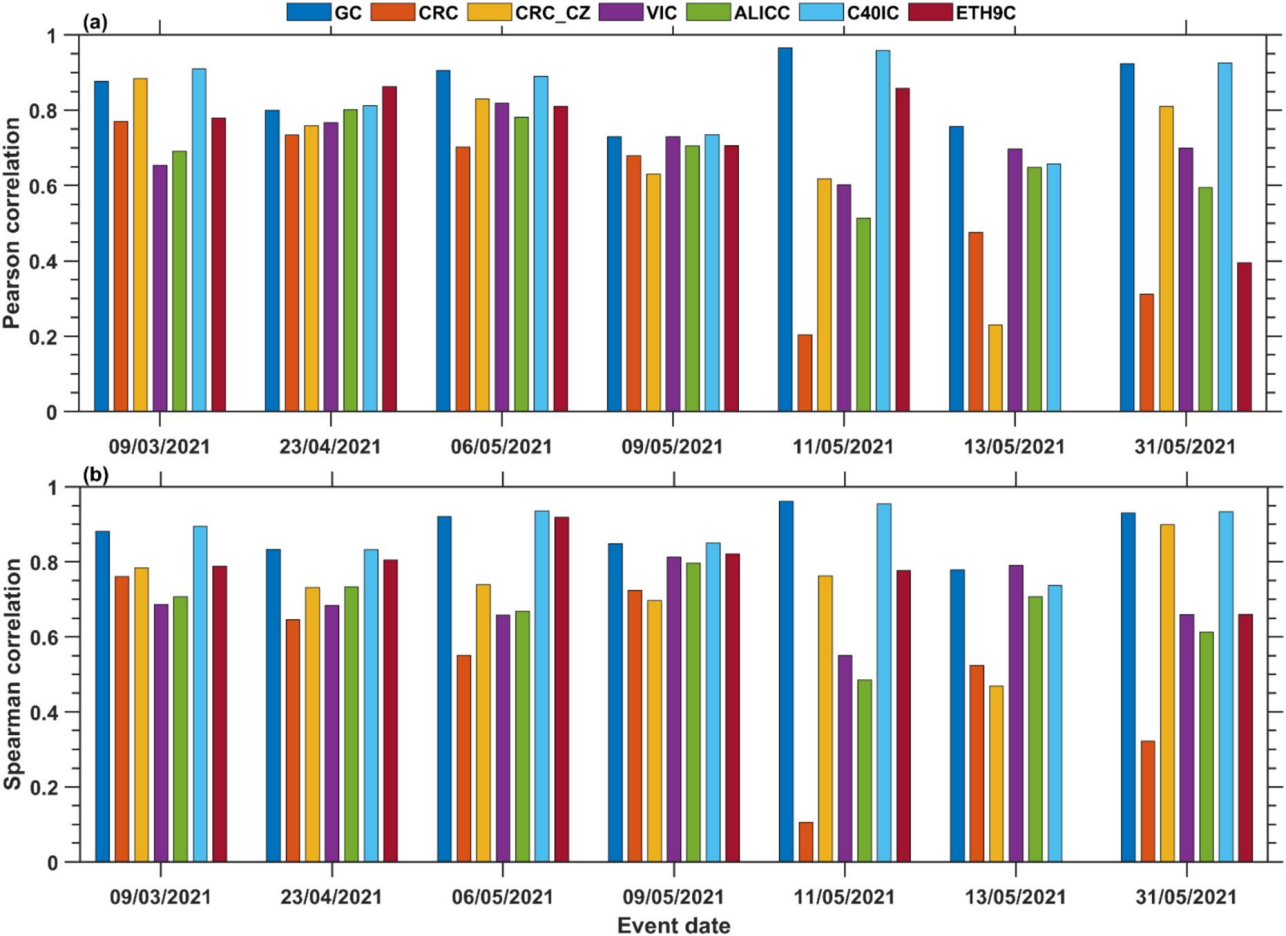
Same as Fig. 9 but for IC lightning.

Event wise correlations between hydrometeor parameters and lightning flash rate for the seven events have been presented in Fig. 10 for IC lightning. Overall correlations are generally higher and more consistent for IC flashes compared to CG flashes across all events and for all parameters. Such as, for GC, correlation values ~ 0.7–0.95 are seen as compared to ~ 0.7–0.85 in case of CG lightning. Tripolar charge structure, formed due to non-inductive charging mechanism and subsequent separation of the charged particles by gravity and updraft, has a main negative charge center located in the middle levels and positive charge center in the upper and lower sections of the storm^39,70^. IC lightning normally initiates between the main negative charge layer and the main upper positive charge layer in the upper level, while CG lightning mainly occurs between the main negative charge layer and ground^71^. Since, IC lightning occurs within the mixed-phase region aloft, it has stronger association with ice microphysics compared to CG flashes.

**Fig. 11:**
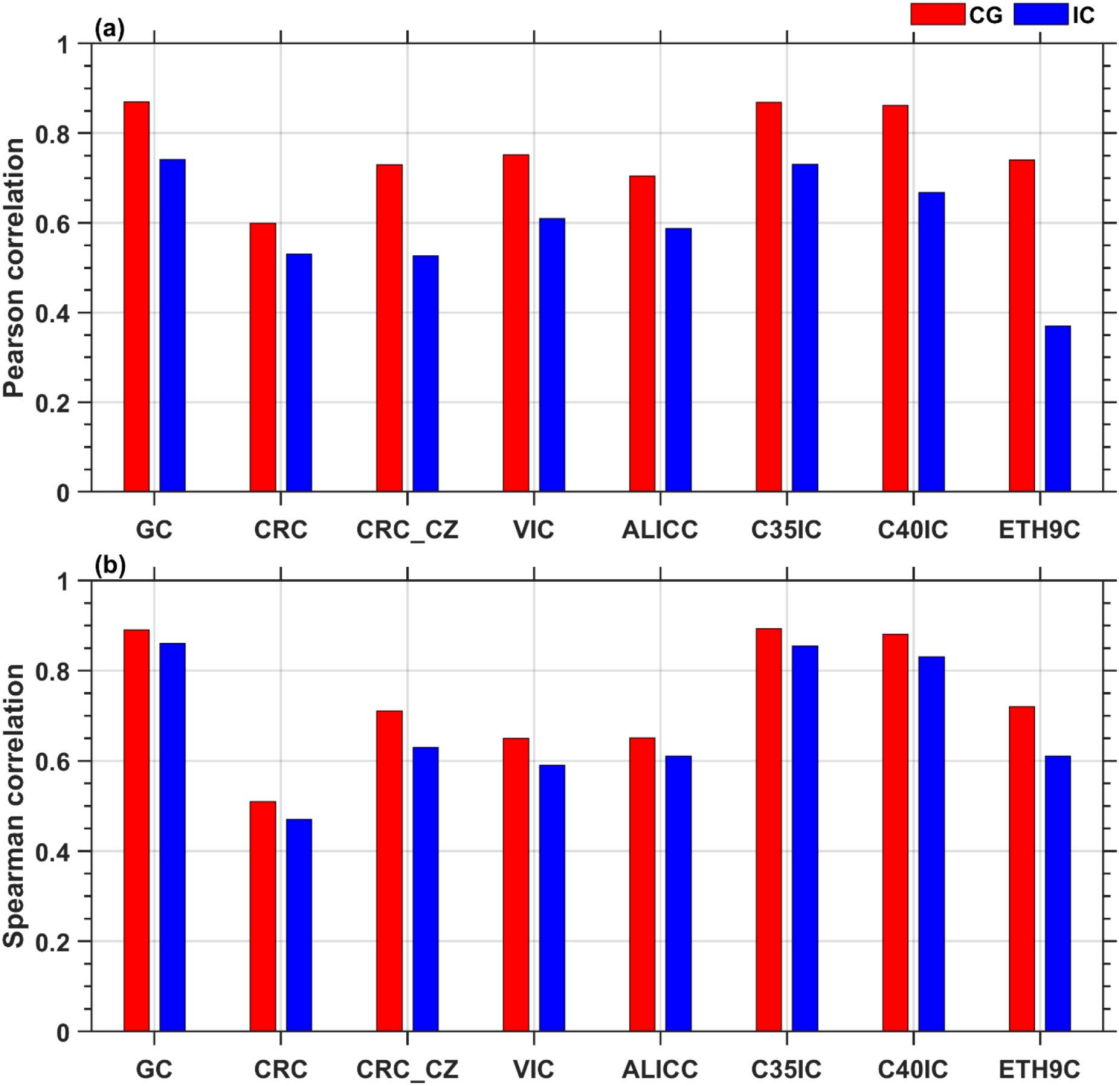
Correlation coefficient values between hydrometeor parameters and flash rates for CG lightning (red bars) and IC lightning (blue bars) lightning. (**a**) Pearson correlation and (**b**) Spearman correlation.

Next, the pooled correlation values i.e., the correlations calculated using all the data from the seven events are shown in Fig. 11. In contrast to the event wise correlation values, the pooled correlations are higher for CG lightning than IC consistently for all the parameters. Such as—for GC, the pooled correlations are 0.87 and 0.74 for CG and IC lightning respectively; for ETH9C, these values are 0.74 and 0.37 for CG and IC lightning respectively. This tells something fundamental about these two types of lightning. Since, the IC lightnings occur in the upper levels of the atmosphere (i.e., mixed phase region), they have more direct relationship with ice hydrometeors present there and this is demonstrated by the higher event wise correlation values for IC. But this relationship with ice hydrometeors varry across different storms, leading to poor correlation when data from different storms are pooled. As mentioned earlier, looking at Fig. 8a we see that for the event 06/05/2021 (yellow circles) and 31/05/2021 (red circles) the data are well correlated for the individual cases, but the slopes of the relationships are very different for the two cases and lead to poor overall correlation. The similar behaviour may be observed for other parameters as well. On the other hand, though the event wise relationships are less strong for CG compared to IC lightning, they fluctuate less across events and lead to better pooled correlations. The smaller inter-storm variability in correlations for CG lightning is most likely due to the fact that CG flashes occur preferentially in mature convective storms with well-developed mixed-phase regions and organized charge structures. Because of these constraints, the relationships between CG flash rate and microphysical parameters remains relatively consistent across events. In contrast, IC lightnings can occur in a wider range of storm stages and regions, including developing updrafts and stratiform regions, where the microphysical and dynamical environments vary substantially between storms. This broader range of conditions introduces greater inter-storm variability in the correlations for IC.

Among the hydrometeor parameters GC, C35IC and C40IC have higher correlation coefficient values with lightning flash rates for both CG and IC lightning. This further shows the importance of graupel in lightning occurrence. VIC is having next highest correlation coefficient values. Another important point to note is that the correlations are higher for GC, C35IC, C40IC, VIC as compared to ETH9C, which signifies stronger relationships of microphysical parameters than storm-intensity parameter with lightning activity. When we compare the correlation values for the CRC and CRC_CZ for the CG type lightning, we can clearly see that it is higher for CRC_CZ (0.73) compared to that of CRC (0.6). This in fact suggests that for the lightning occurrence, the ice crystals in the charging zone are the most relevant ones. This result is in line with the observations of charge structures of thunderclouds by Stolzenburg et al.^40^, in which the authors have shown that inside thunderclouds, the positive (+ Ve) charges are mainly observed at levels above − 25°C and as per the non-inductive charging theory these positive charges are carried by ice crystals.

### Time lag correlation analysis for nowcasting possibilities

**Fig. 12:**
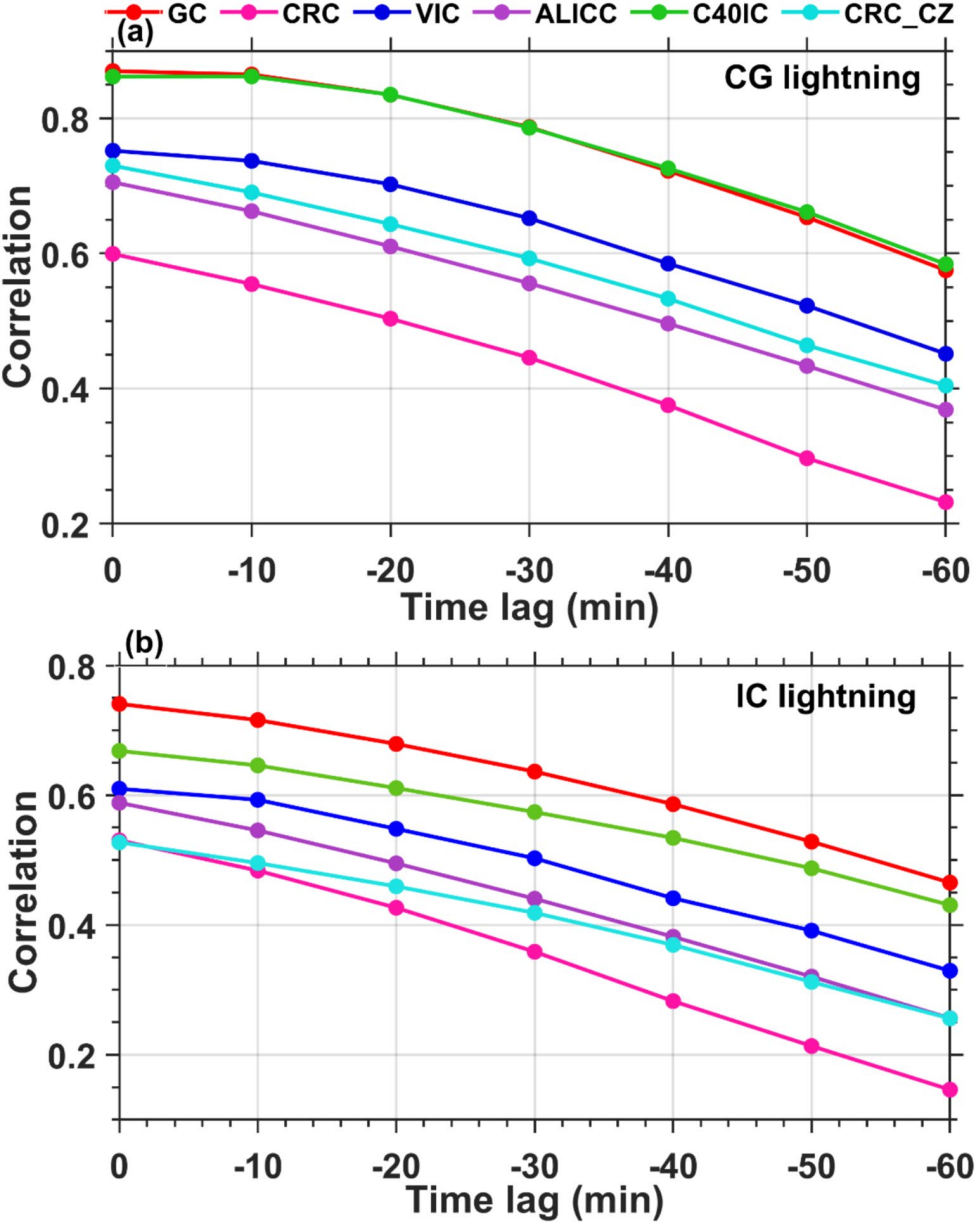
Time lag correlation between hydrometeor parameters and lightning flash rates for (**a**) CG lightning and (**b**) IC lightning.

Since, from the previous analysis we have seen a strong correlation between hydrometeor parameters and lightning flash rates, here we explore the possibilities of lightning nowcasting using these hydrometeor parameters. For this we have done a time lag correlation analysis. Basically, we have calculated the same correlations as we did in the previous section, but here we take the values of the hydrometeor parameters at some earlier times (i.e., time lag) compared to that of the lightning flashes. We have considered time lag up to 60 min. The results of the analysis are presented in Fig. 12. We have done this for both CG (Fig. 12a) and IC lightning (Fig. 12b). As expected, the correlation values decrease with higher time lags. GC and C40IC are having the higher correlation values for CG lightning for all time lags. They are having almost similar values at all time lags. But for IC lightning, GC is having higher correlation compared to C40IC at all time lags, which suggests that IC lightnings are controlled by smaller ice crystals which do not produce high (> 40dBZ) reflectivity values. VIC is having the next highest correlation values for both CG and IC lightning at all time lags. If we compare the correlation values among ALICC, CRC and CRC_CZ for the IC type lightning, we observe some interesting features. At zero time-lag, ALICC is having the highest correlation among them, but as we move to higher time lags, the correlations values for ALICC and CRC_CZ come closer to each other. On the other hand, even though initially, CRC and CRC_CZ are having similar correlation values, as we move to higher time lags, CRC_CZ is having higher correlation values compared to that for CRC. This means that for IC lightning, all ice particles play certain roles and the ice crystals at charging zone are more important when it comes to lightning flash. Altogether, GC, C40IC and VIC are showing very good lag correlation coefficient values (greater than or very close to 0.6) even up to 40 min of lag. This time lag correlation analysis suggests that it is very much plausible to use these hydrometeor parameters as precursors for lightning nowcasting.

**Fig. 13:**
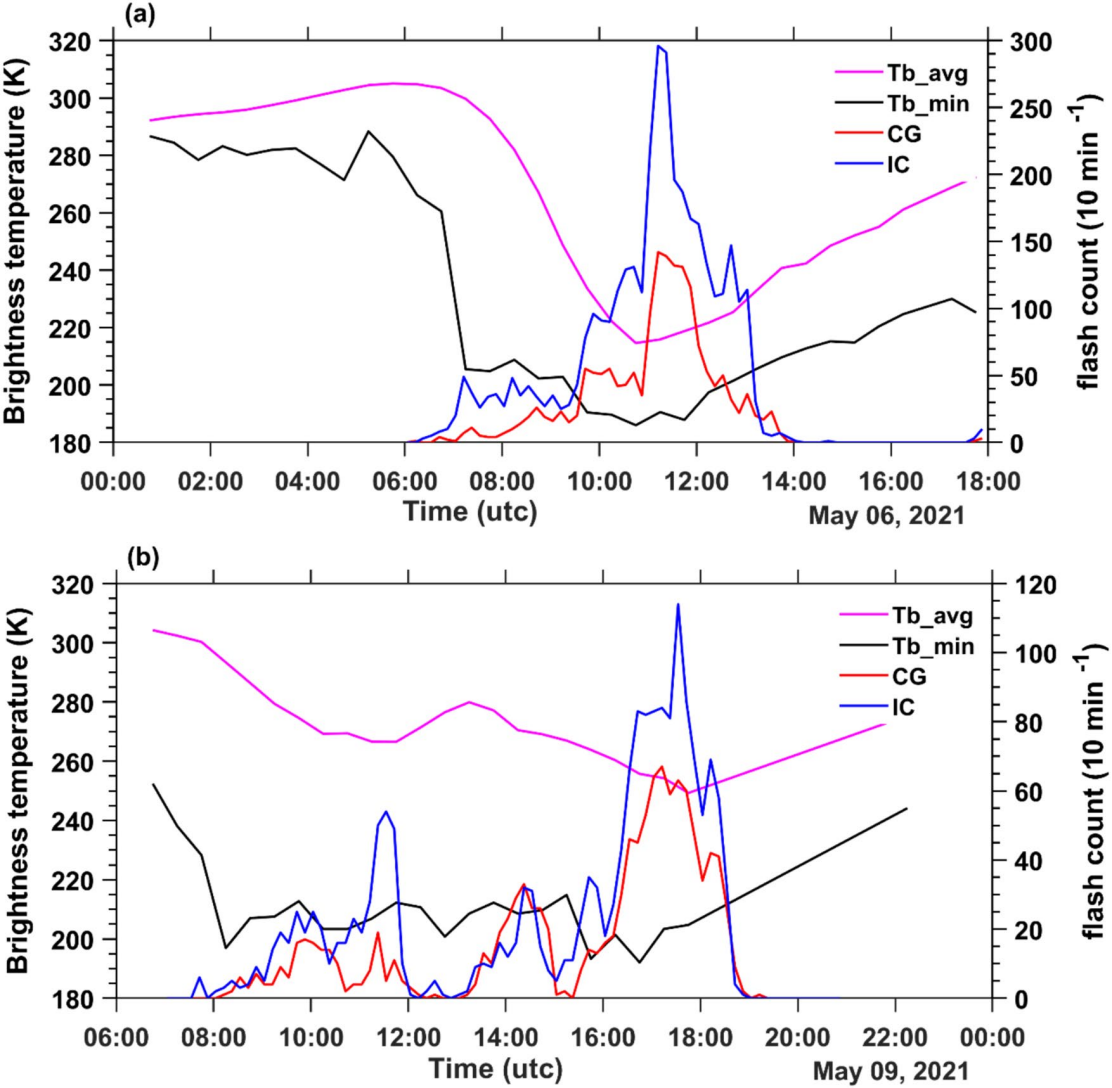
Time series of cloud top brightness temperature from INSAT-3DR satellite and lightning flash rates for CG (red) and IC (blue) type of lightning for thunderstorms on (**a**) 06-May-2021 and (**b**) 09-May-2021. Average brightness temperature (pink) and minimum brightness temperatures (black) are shown.

We have extended this nowcasting possibility further with the use of cloud top infrared brightness temperature data obtained from INSAT-3DR satellite. In Fig. 13, we have shown the temporal evolution of brightness temperatures and the flash rates for CG and IC lightning for the thunderstorms on 06-May-2021 and 09-May-2021. Time series of average brightness temperature (Tb_avg) and minimum brightness temperature (Tb_min) over the study region (i.e., 200 × 200 km box centred at DWR location) are shown. For the thunderstorm on 06-May-2021, we see a sharp decrease in the value of Tb_min well before the time of peak lightning activity. Similar features are observed for the thunderstorm on 09-May-2021 as well. The sharp decrease in brightness temperature signifies the development of deep clouds due to the presence of strong updrafts and resulted in subsequent strong lightning activity. It is to be noted that the lightning activity for thunderstorm on 06-May-2021 was much stronger than that of the thunderstorm on 09-May-2021. Such sharp decrease in the brightness temperature during a pre-monsoon thunderstorm over Thiruvananthapuram, Kerala has been observed by Jash et al.^50^. Time lag correlation up to lag of 90 min, for Tb_min and Tb_avg with flash rates for CG and IC lightnings are shown in Table 2. The negative sign in the correlations indicate inverse relationship, i.e., a decrease in brightness temperature corresponds to an increase in the flash rate. For Tb_min, correlation values greater than 0.5 is seen up to 60 min of lag for CG lightning. For IC lightnings, less correlation is seen compared to CG. Higher correlations for CG flashes may be because, CG lightnings mostly occur when deep clouds develop due to strong updrafts, leading to stronger electric field development required for CG flashes. These findings suggest that cloud top brightness temperature in conjunction with the storm microphysical parameters could be used as proxies for lightning nowcasting.

**Table 2:** Lag correlation values for minimum and average brightness temperature with flash rates for CG and IC lightning.

Lag time (minute)	Pearson correlation				Spearman correlation			
	Tb_avg		Tb_min		Tb_avg		Tb_min	
	CG	IC	CG	IC	CG	IC	CG	IC
30	− 0.45	− 0.36	− 0.51	− 0.39	− 0.24	− 0.24	− 0.60	− 0.48
60	− 0.33	− 0.23	− 0.45	− 0.32	− 0.05	− 0.07	− 0.51	− 0.36
90	− 0.18	− 0.10	− 0.35	− 0.23	0.11	0.10	− 0.34	− 0.21

## Summary and conclusions

The present study focuses on the relationship between ice hydrometeors in thunderclouds and associated lightning activity as suggested by the non-inductive charging theory, which states that the charge separation due to rebounding collision between graupel and ice crystals is the dominant charging mechanism of thunderclouds. To investigate this, we have identified the hydrometeor types within the thunderclouds by applying a fuzzy-logic based hydrometeor identification algorithm on a C-band polarimetric DWR data and estimated several ice hydrometeor related parameters. Also, we have obtained lightning flash rates for both CG and IC flashes from ILLN data. Relation between hydrometeor parameters and lightning has been analysed in terms of similarities in spatial patterns, temporal evolution and correlation values. A time lag correlation analysis has been performed to assess the usefulness of these microphysical parameters as proxies for lightning nowcasting. Another parameter related to storm intensity has also been considered to assess the strength of the relationships for microphysical parameters compared to the storm-intensity parameter. Finally temporal evolution of cloud top infrared brightness temperature from INSAT-3DR satellite has been studied to strengthen the possibilities of nowcasting of lightning. This study has been done over Delhi and its surrounding regions using data from seven prominent thunderstorm events during pre-monsoon season of 2021. The major findings of the study are as follows.

1. A strong association in spatial patterns of graupel and lightning occurrence (both for CG and IC type) during thunderstorms are seen. For more than 95% of CG and IC flashes there exist graupel within 1 km radius. For more than 80% and 70% of graupel locations IC and CG flash have been detected within 4 km radius.
2. Time evolution of ice hydrometeor related parameters (such as GC, CRC, VIC, C35IC) and lightning flashes during the life of the thunderstorms are similar.
3. Event wise correlations are stronger for IC flashes compared to CG flashes as IC flashes occur in the mixed phase layer and more directly linked to the ice-hydrometeors.
4. Correlations for pooled data across storms are higher for CG flashes for all parameters. Higher inter-storm variability of the relationships leads to poorer correlations, in case of IC flashes.
5. Higher correlations for GC, C40IC, VIC compared to ETH9C represent stronger relationships between storm microphysical parameters and lightning than between storm-intensity parameters and lightning.
6. Lag-correlations between ice hydrometeor parameters and lightning flash rates are quite good (greater than 0.6) up to 40 min of lag, particularly for GC, C40IC and VIC. This suggests that GC, C40IC and VIC may be used for nowcasting of lightning up to 40 min in future.
7. Time evolution of cloud-top brightness temperature from INSAT-3DR satellite and lightning flash rate shows that decrease in brightness temperature precedes the occurrence of lightning by more than an hour. It suggests that cloud-top brightness temperature may be used alongside the above hydrometeor parameters as precursor for lightning occurrence.

This study utilizes data from seven thunderstorm events. There is a need to incorporate more events to solidify these findings further. Also, we need to take forward the nowcasting analysis using these hydrometeor parameters and brightness temperature data and derive some analytical relationship so that it could be applied for giving warning of lightning for the society. One promising way could be to train appropriate deep learning models with these hydrometeor parameters and brightness temperature as input and the lightning flash rate as the target. Thus, we can obtain a trained deep learning model for prediction of lightning.

## Data Availability

The Doppler weather radar (DWR) data used in this study are available online (on request) through the website https://radarapi.imd.gov.in/.
